# Automatic Switching of Electric Locomotive Power in Railway Neutral Sections Using Image Processing

**DOI:** 10.3390/jimaging10060142

**Published:** 2024-06-11

**Authors:** Christopher Thembinkosi Mcineka, Nelendran Pillay, Kevin Moorgas, Shaveen Maharaj

**Affiliations:** 1 Transnet, 121 Jan Moolman Street, Vryheid 3100, South Africa; christopher.mcineka@transnet.net; 2 Department of Electronic and Computer Engineering, Durban University of Technology, Steve Biko Campus, Durban 4001, South Africa; kevinm@dut.ac.za (K.M.); shaveenm@dut.ac.za (S.M.)

**Keywords:** computer vision, neutral section, image processing, Circular Hough Transform, histogram of oriented gradient, classifier

## Abstract

This article presents a computer vision-based approach to switching electric locomotive power supplies as the vehicle approaches a railway neutral section. Neutral sections are defined as a phase break in which the objective is to separate two single-phase traction supplies on an overhead railway supply line. This separation prevents flashovers due to high voltages caused by the locomotives shorting both electrical phases. The typical system of switching traction supplies automatically employs the use of electro-mechanical relays and induction magnets. In this paper, an image classification approach is proposed to replace the conventional electro-mechanical system with two unique visual markers that represent the ‘Open’ and ‘Close’ signals to initiate the transition. When the computer vision model detects either marker, the vacuum circuit breakers inside the electrical locomotive will be triggered to their respective positions depending on the identified image. A Histogram of Oriented Gradient technique was implemented for feature extraction during the training phase and a Linear Support Vector Machine algorithm was trained for the target image classification. For the task of image segmentation, the Circular Hough Transform shape detection algorithm was employed to locate the markers in the captured images and provided cartesian plane coordinates for segmenting the Object of Interest. A signal marker classification accuracy of 94% with 75 objects per second was achieved using a Linear Support Vector Machine during the experimental testing phase.

## 1. Introduction

**Figure 1:**
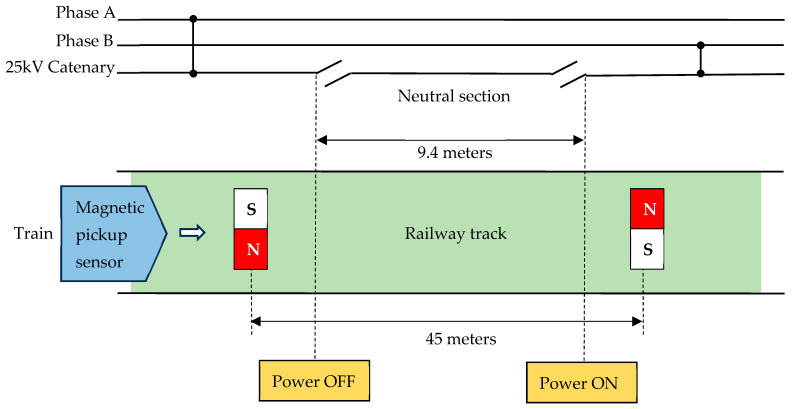
Neutral section switching configuration at Transnet Railway.

Transnet is a South African state-owned company, and National Freight Rail is one of the divisions with approximately 8000 km of electrified railway overhead lines. The electrified lines are split into 3 kV DC (861 km), 25 kV AC (2516 km), and 50 kV AC (4621 km). In a 25 kV AC traction system, it is common to find the neutral sections (NS) installed along the railway overhead lines. The purpose of a NS is to separate two single phases from shorting circuiting, which avoids tripping of the main power substations. There are currently three conventional railway NS switching schemes, namely, ground switching, pole switching, and onboard switching. The method of ground switching is based on vacuum circuit breakers (VCBs), which are installed on the ground, and sensors are used to detect the presence of a train. When a train is detected, it operates the VCBs to automatically switch power to the train while it traverses through the NS. Han et al. [1] developed a system that uses a ground substation, whereby axle counters on the ground detect the position of the train and subsequently enable the substation to switch breakers. The system uses mechanical VCBs, which require frequent maintenance due to the reduced life span caused by the high voltage switching. Ran et al. [2] suggested replacing the mechanical switches with Silicon Controlled Rectifiers (SCRs); however, this would require additional firing control circuitry that adds further complexity to the system. Transnet Freight Rail implements the onboard switching scheme to switch electric locomotives when traversing through a NS [3]. This scheme includes induction magnets that are installed in-between the railway tracks at opposite ends of the NS and an onboard magnetic sensor installed underneath the electric locomotive. When an electric locomotive passes through one set of induction magnets, the onboard magnetic sensor is activated, which subsequently initiates the onboard system to trigger the VCBs to remove power from the locomotive. When the locomotive has traversed past the NS, a second set of magnets switches the VCBs, thereby switching the locomotive ‘on’ with a different single-phase voltage supply. Figure 1 illustrates a typical Transnet railway NS installed at a length of 9.4 m on the overhead catenary line with two separated 25 kV AC single phases. A set of induction magnets, each having North and South polarities, is installed at the opposite ends of the railway track, 45 m apart.

A major drawback to this approach is that there is a high cost incurred during the installation and maintenance of the railway magnets, which are prone to theft. Furthermore, deterioration of the magnetic field strength may result in the locomotive sensor failing to detect a NS changeover. To circumvent the problems associated with this switching technique, an alternate methodology is to replace the railway induction magnets with visible signboards and an image identification system. A camera can then be mounted on the locomotive to capture the stationary railway marker images. Image recognition can be employed to initiate the switching sequence at either end of the NS. The idea of using image recognition for automatic NS switching was proposed by Chen et al. [4]. In their work, a simplistic edge detection technique was used to compare the diagonal lengths of existing railway markers. However, their system was not tested in rainy and foggy weather conditions. This approach was further extended by Mcineka and Reddy [5] and Mcineka and Pillay [6], where the marker image is pre-processed and followed by localization of the Region of Interest (RoI). The RoI is then segmented into Objects of Interest (OoI), where a machine learning algorithm is employed to perform the classification of each OoI. In this article, additional insights into this methodology are provided, and key findings are discussed.

The article is structured as follows: A discussion of related work on computer vision applications in the railway industry is given in Section 2. Section 3 describes the proposed computer vision model used for NS switching. Section 4 provides the system performance of optimal parameter settings and comparative tests for the different machine learning models for the selected application. Section 5 provides the conclusion of the study with recommendations for improvement.

## 2. Related Literature for Object Detection in the Railway Industry

Over the last few years, deep learning methods have shown their adept ability to outperform traditional methodologies in several fields, for instance, autonomous driving and road sign recognition [7,8]. Notably, computer vision is a prominent discipline that has rapidly evolved and progressed using various rich deep learning techniques. Since the focus of this work is to apply computer vision methods to detect unique railway signal markers, image processing and object detection methods will fundamentally play a vital role in the successful implementation of NS. Primarily, object detection is the process of detecting instances of certain classes in a digitized image. A common approach in the object detection framework is to create an adequate set of candidate images for training and validation purposes. Various image preprocessing techniques are usually employed to derive distinctive object characteristics that focus on the image ROI and subsequent OoI.

It should be noted, however, that when examining related works about railway computer vision applications [9,10,11,12,13,14,15,16,17,18,19,20,21,22,23,24,25,26,27,28], there is still much room left for the field to mature. Furthermore, it is stressed that existing algorithms and approaches are not universal, since railway signage differs in various countries around the world. The primary variations exist to address specific applications, which may not be universally applicable to all railway systems.

Early developments in the field of railway sign identification were similar to the recognition approaches tailored to road signs, a problem for which many solutions have been proposed in the literature. Traditional approaches made use of color segmentation and template matching techniques, which were highly susceptible to varying illumination, changing weather conditions, perspective distortions, motion blur, and image rotation. In the research conducted by [9], a Scale Invariant Feature Transform (SIFT) method was used to circumvent these issues and extract distinctive features from the captured greyscale railway sign images. Their chosen target for the experiments was selected as the Japanese ‘slow-speed-notifying signal’ sign board. The proposed approach was built upon clusters of modified SIFT features that were able to learn specified features of the selected target sign and achieved a recognition rate of 90%. An alternate approach was given by [10], whereby the authors developed their own algorithm to detect a selected class of ‘W11p’ railway signs. They simplified the processes of localization, segmentation, and recognition using a backpropagation Artificial Neural Network (ANN). Their method included an interactive approach for generating the training dataset, enabling diverse colour pixel inclusion, and using a full spectrum ANN response for automatic threshold adjustment based on segmentation and recognition results. The system achieved 90% effectiveness at detecting ‘W11p’ signs and approximately 97% effectiveness at classifying them. However, a relatively small sample set of 71 railway signs was used to train the model.

More recently, a comprehensive review of vision-based on-board obstacle detection in railways was given by [11]. They also confirm that development in this field is far less established, as evident by the significantly smaller number of published related works in comparison to road sign recognition. Furthermore, there is a need for additional computer vision research and development, particularly in the railway transportation industry. The authors point out that on-board obstacle detection in railway systems can be classified into two main categories, namely, traditional computer vision and Artificial Intelligence (AI) methods. In traditional computer vision systems, ‘hand-crafted’ features such as edge detection, shape descriptors and threshold segmentation are utilized in the object recognition process. In contrast, AI-based methods, in particular deep learning, use Machine Learning (ML) and CNN techniques for end-to-end learning of features extracted directly from the captured images. In addition, the authors also allude to limited publicly available datasets. They suggest that this may be a contributing factor to the fewer published works in the literature for railway image detection and classification. The dataset ‘RailSem19’ was introduced as an openly available data source specifically developed for semantic railway scene understanding. It contains images from the viewpoint of a train and is specific to a variety of tasks that include the classification of trains, switch plates, and other objects typically found in railway scenarios. However, the sample dataset does not contain any anomalies or obstacles. In this study, the development of the proposed model is based on custom-made railway images of unique switching markers mounted alongside the railway line. The image acquisition process is subsequently described in Section 3.1.

Other railway applications that use AI methods for image recognition include rail track detection [12,13,14,15], obstacle detection [16,17,18,19,20], and distance estimation [21,22,23]. In the field of railway management systems, asset mapping is an important consideration. This field has the potential to be greatly improved using computer vision approaches. Due to the lack of available data, [24] proposes the use of a Faster R-CNN (Region-based Convolutional Neural Network) approach to address autonomous railway asset detection. They achieved a 79.36% accuracy on the detection of railway signals and a mean average precision (mAP) of 70.9% using their dataset. Their system has the potential to be improved since the authors claim that the results were compromised by a high degree of similarity across the different classes and relatively small object sizes in the low-resolution images that were captured.

More recently, Ref. [25] discusses the importance of automated detection and recognition of traffic signals in railway systems, especially for mainline locomotives, where autonomous driving is still challenging due to the complex nature of the environment. The authors introduce a deep learning method using the You Only Look Once (YOLOv5) architecture for detecting and recognizing wayside signals, including a heuristic for identifying blinking states. The system was trained on a curated version of the ‘FRSign’ dataset emanating from the French Railways, thereby enabling real-time recognition under various conditions, such as rainy and nighttime environments.

Another important consideration is railway track circuit signal object detection. In the research conducted by [26], they propose a two-phase detection algorithm to monitor the status lights of the track circuit signals in the control room. Their computer vision monitoring system effectively provides important information for humans to conduct work on the railway track, thus enhancing safety when driving the locomotive.

A safe level crossing at the intersection of a railway line with a pedestrian footpath, road, or bridge intersection is another area that has been explored using deep learning techniques [27,28]. Level crossings in particular pose many safety challenges and are a significant risk to the public. Traditional sensing systems often rely on a single sensor, which may not provide sufficient information for effective decision-making and automation. In a previous study [27], the authors proposed a Closed-Circuit TeleVision (CCTV) system with integrated deep learning object detection algorithms to track specific targets such as pedestrians, vehicles, and bicycles to assist with the prevention of accidents and fatalities. The authors suggest that the computer vision system can be further enhanced using a radar device to act as a fail-safe mechanism. Similarly, in the work presented by [28], the authors propose an intelligent safety system that combines object detection and classification methods using various image processing inputs related to railway crossings. The system employs a Graphical Processing Unit (GPU) for accelerated image processing and deep learning ANNs to autonomously detect potential risky situations with vehicle and pedestrian trajectory tracking in real-time. The system can send critical safety information to a central server for further processing and notification to railway operators and relevant emergency services. Field-based results using the YOLOv3 tiny model achieved an average recall of 89%, indicating the system’s efficiency in detecting objects and potential accidents at railway crossings. As can be seen from the literature survey, there is a diverse range of applications in which computer vision has been utilized to solve various problems in the railway industry. To the authors knowledge, there is still a gap in the literature that addresses the use of image processing specific to NS voltage changeover. Therefore, the main contributions of this paper are summarized as follows:

- We suggest the replacement of the conventional electro-mechanical system used for switching traction supplies with a computer-based vision system. The advantage of such a system would be that it would reduce maintenance costs and enhance the reliability of the system. Visual detection has the potential for high accuracy and rapid automation.
- The introduction of visual markers along the railway line as triggers for NS would enable precise and automated control of the vacuum circuit breakers within the locomotive.
- Employing the Circular Hough Transform shape detection for image segmentation enhances the accuracy of locating the markers in the captured images.
- The implementation of image classification using a Histogram of Orientation Gradient technique and training a Liner Support Vector Machine algorithm for target image classification are novel approaches in this context.

## 3. Methodology for Neutral Switching Using Image Detection

**Figure 2:**
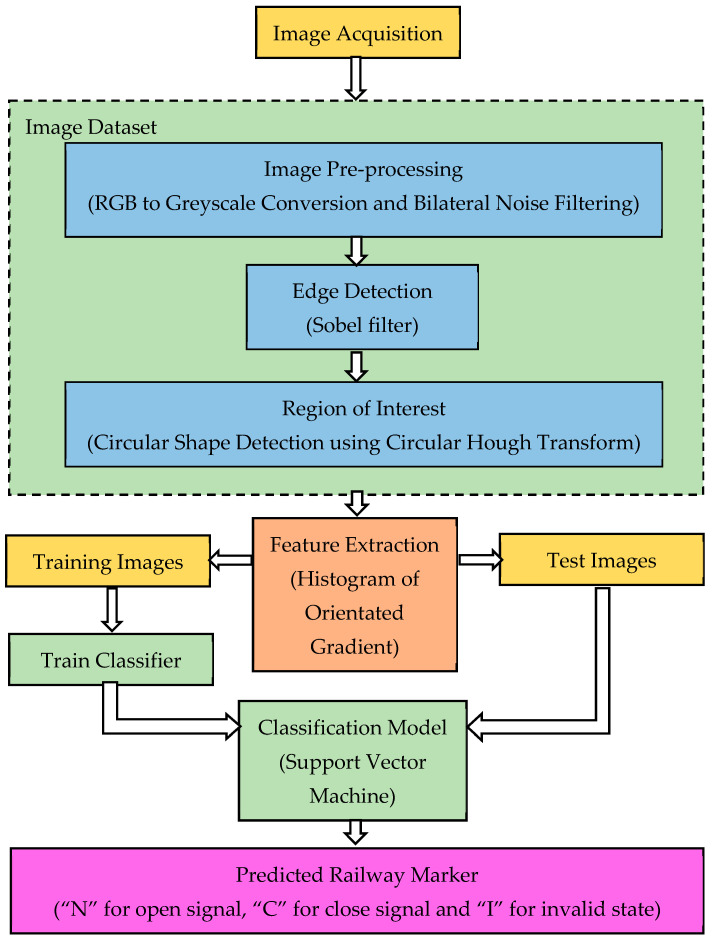
Proposed architecture of the computer vision model for railway NS.

Computer vision can be defined as the perception of objects through a camera and a computer. The camera acquires an image, and a computer processes the image, and then classifies or interprets what the image contains. The data for this research focused on two markers located at strategic points along the NS railway. An open signal is defined as “N”, and a close signal is denoted by “C”. The label “I” defines an invalid model output state. The selected criteria for the markers were pre-defined, such that they needed to be circular in shape, have a white foreground with a black background, and conform to the South African National Standard for safety signs (SANS1186). Additionally, it should be clearly visible at a certain distance during varying lighting and motion blur situations. Figure 2 presents an overview of the proposed computer vision system. Each stage is described in the subsequent sections.

### 3.1. Image Acquisition

**Figure 3:**
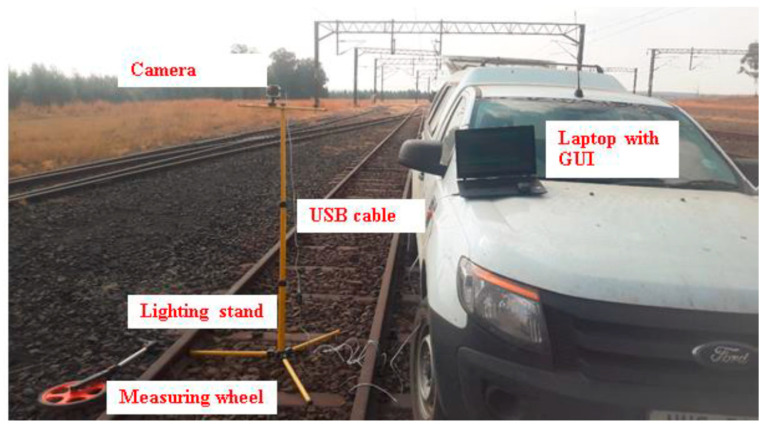
Experimental image capturing setup.

**Figure 4:**
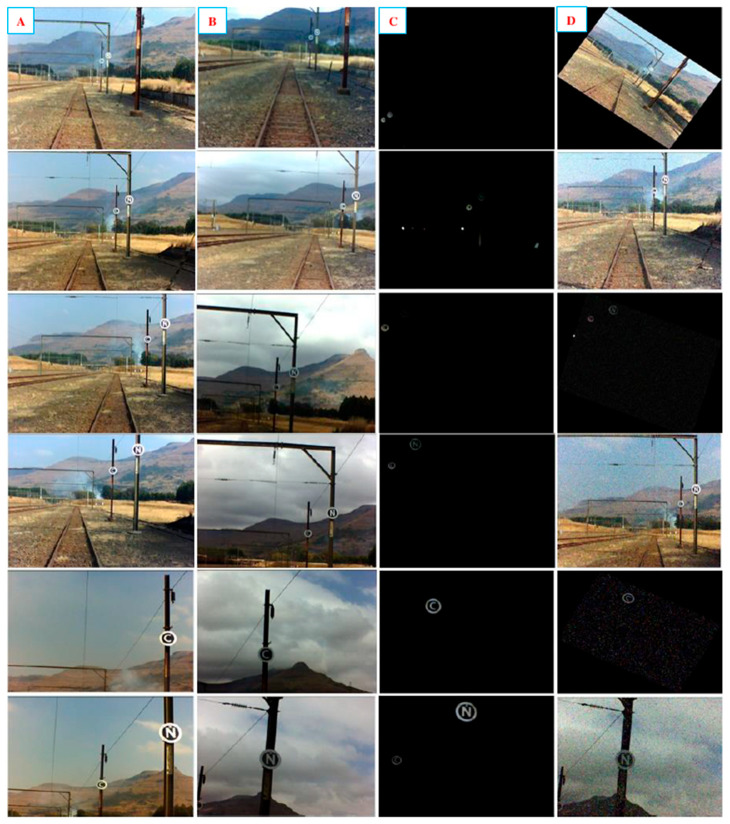
Dataset of the captured images. Column (**A**) represents images on a sunny day, Column (**B**) represents images on a cloudy day, Column (**C**) shows images captured at night, Column (**D**) shows images captured with random noise and rotation. Capture distance from Top to Bottom Row indicates images captured at distances of 45 m, 30 m, 25 m, 14 m, and 10 m.

Before training and testing the model, a dataset was required to store the acquired images for further processing. Figure 3 shows the initial setup during image acquisition for obtaining a dataset. A lighting stand was used to simulate the height of an electric locomotive, where the camera would be mounted to capture the markers. A measuring wheel was used to measure the capture distance, ranging from 10 m to 45 m away from the markers. A laptop with an Intel^®^ Core processor (i5-10210U) running at 1.60 GHz with 16 GB of RAM was used to host a Graphic User Interface (GUI) application. The purpose of this was to conveniently capture and store the images based on the captured distance, noisy or distorted frames caused by the train motion, weather conditions, and the time of day (day or night). Furthermore, saving these images into a specific folder allows you to easily split the images into training and testing images. Images were captured using a Charged Coupled Device (CCD) camera at a resolution of 640 × 480. In [29], the selection of a CCD camera over a Complementary Metal Oxide Semiconductor (CMOS) is motivated. Figure 4 illustrates the captured images for the varying conditions. The compiled dataset comprised a total of 550 images of which an additional 104 were negative or invalid images. Finally, the dataset was split between 70% and 30% and used for training and testing purposes, respectively.

### 3.2. Image Pre-Processing

#### 3.2.1. RGB to Greyscale Conversion

The images are acquired in the red (*R*), green (*G*), and blue (*B*) channels of the RGB colour space, and image processing was then applied to convert the images into a greyscale colour space. The motivation for this is to convert the RGB image (where each channel can be defined by m×n×3 array) into a greyscale image that only has one channel that can range from 0 to 255 (where 0 represents absolute white and 255 represents absolute black color) for an 8-bit colour system [30]. To convert an RGB image to a greyscale image, the updated colour space is defined by Equation (1):

(1) $$Y=\left(0.299\times R\right)+\left(0.587\times G\right)+\left(0.114\times B\right)$$

where Y denotes the resulting luminance.

#### 3.2.2. Bilateral Noise Filter

These acquired images undergo a noise-filtering stage to remove noise and background artefacts. The denoising of images is an important part of image processing to eliminate the noise embedded in the image [31]. In this study, the bilateral filter is utilized for noise removal. The primary motivation for its use was based on its ability to smooth noisy images while preserving edges. The Bilateral noise filter used in the pre-processing phase is given as follows:

(2) $$BF\left[I_{p}\right]=\frac{1}{W_{p}}\sum_{q\in S}G_{\sigma_{s}}\left(\left\|p-q\right\|\right)G_{\sigma_{r}}\left(\left|I_{p}-I_{q}\right|\right)I_{q}$$

(3) $$W_{p}=\sum_{q\in S}G_{\sigma_{s}}\left(\left\|p-q\right\|\right)G_{\sigma_{r}}\left(\left|I_{p}-I_{q}\right|\right)$$

Equation (2) defines the filtered image, with each pixel modified by applying the bilateral filter. To ensure that the pixel weight sum does not exceed one, a normalization factor weight $W_{p}$ is used. Equation (3) defines this normalization factor that is assigned to the neighboring pixel p and a denoise pixel located at q coordinates. The variables are defined as:

- $I_{q}$: Original image value at pixel position q.
- $I_{p}$: Filtered image value at pixel position p.
- $W_{p}$: Spatial and range weights of the neighboring pixel p.
- p: Coordinate of the neighbouring pixel to be filtered.
- q: Coordinate of the current pixel to be filtered.
- S: Window centered in q, so p∈S defines another pixel.
- $G_{\sigma_{s}}$: Spatial Gaussian weighting (for smoothing).
- $G_{\sigma_{r}}$: Range Gaussian weighting (preserves contours).

Algorithm 1 describes the pseudocode to implement the image preprocessing stage.
**Algorithm 1.** Image conversion and filteringInput: Greyscale marker images Output: Grayscale noise-filtered images
 Declare variable *(numberOfImages)* Find the number of images in the dataset: store in *numberOfImages* for each image in the dataset ≤ *numberOfImages,* do    Read each image    if an image is in RGB colour space, do     Convert to greyscale using Equation (1)    else      Do nothing, already in greyscale    end if Apply a bilateral filter to remove noise using Equation (2) end for

### 3.3. Edge Detection Using the Sobel Operator

There are several common edge detection algorithms in the literature, namely Sobel, Canny, Laplacian of Gaussian (LoG), and Roberts. The basic operation of an edge detection algorithm is applying a convolution mask called a kernel to an image. The kernel is convolved into an image to identify and locate discontinuities. These discontinuities define the boundaries of objects in an image and are detected by finding abrupt changes in pixel intensity. In this study, the Sobel operator was employed for edge detection of grayscale images. The selection was justified by its performance when compared with other operators using the same dataset [3]. Subsequently, most of the background objects were removed while preserving the predominant edges of the markers.

Equations (4) and (5) define a Sobel operator for a 3-by-3 mask, where $G_{x}$ identifies and locates horizontal gradients, while $G_{y}$ represents the vertical gradients. Equation (6) determines the edges by computing the absolute gradient magnitude ($\left|G\right|)$.

(4) $$G_{x}=\left[\begin{array}{ccc} -1 & 0 & 1\\ -2 & 0 & 2\\ -1 & 0 & 1 \end{array}\right]$$

(5) $$G_{y}=\left[\begin{array}{ccc} \;\;\;\;\;1\; & \;\;\;2 & \;\;\;1\\ \;\;\;\;0 & \;\;\;0 & \;\;\;0\\ -1 & -2 & -1 \end{array}\right]$$

(6) $$\left|G\right|=\sqrt{{G_{x}}^{2}+{G_{y}}^{2}}$$

The direction or angle (θ) of the edges is computed by applying Equation (7):

(7) $$\theta =\tan^{-1}\frac{G_{y}}{G_{x}}$$

A non-maximum suppression can then be applied to trace along the edge direction. The latter is carried out to suppress any pixel value that is not considered an edge.

### 3.4. Locating the Region of Interest

**Figure 5:**
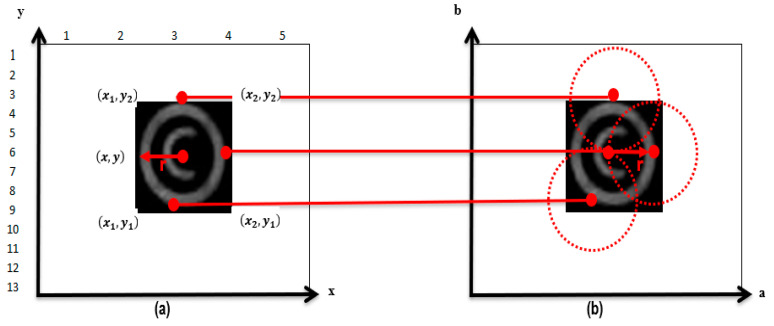
CHT transformation. (**a**) x, y Cartesian plane. (**b**) a,b parametric space.

A Circular Hugh Transform (CHT) algorithm is then applied to the newly generated image after applying the Sobel operator. We apply a CHT algorithm to delineate the coordinates of the RoI so that the OoIs of each marker can be extracted. The CHT, being a shape-detecting algorithm, was found to be the best choice since it detects circular shapes and is well suited to this application. To increase the efficiency of detecting more markers, a minimum diameter of 10 pixels and a maximum diameter of 60 pixels was chosen. The primary reason for minimum and maximum-diameter pixels is due to varying capture distances. Images captured at 10 m will use 60 pixels due to the larger diameter of the marker. Conversely, at 45 m, the image’s diameter is 10 pixels. These values allow for the CHT algorithm to have a radius that has a minimum and maximum value. Figure 5 illustrates how the CHT algorithm is effectively used to detect circular shapes within the image. The CHT algorithm transforms a circle in the image from the two-dimensional (x, y) cartesian plane to a three-dimensional parameter space $\left(a,\;b\right)$. This approach transforms the (x, y) into parametric space, which contains the circles radius (r) as defined by Equation (8):

(8) $$r=\sqrt{{\left(x-a\right)}^{2}+{\left(y-b\right)}^{2}}$$

The transformation of OoI which is a circle in the dataset from (x, y) plane to a parametric space (x, y, r) is illustrated in Figure 5.

To obtain the coordinates to crop the OoI’s, a bounding box approach is applied. Equations (9)–(14) was used to calculate the size of the box:

(9) x=a+rcos⁡θ

(10) y=b+r sin⁡θ

(11) x1=x−r

(12) x2=x+r

(13) y1=y−r

(14) y2=y+r

The steps given in Section 3.3 to Section 3.4 are summarized in the pseudocode as illustrated by Algorithm 2.
**Algorithm 2.** Segmentation an RoI extractionInput: Greyscale noise-filtered images (Algorithm 1) Output: Cropped images with OoI’s (markers)
 Vector variables *centres*, *radii* and *circlesFound* for each greyscale-filtered image, do   Apply the Sobel operator using Equations (4)–(7)   Find the centres and radii using Equation (8)   Compute *circlesFound* in each image with radii.   for *circlesFound* ≥ 1, do      Get the radius of each circle      Calculate coordinates: Equations (9)–(14)      if (circle centre − radius) < 0, do       if *x*1 ≤ 1, do       *x*1 = 1       else *x*1 = radius − centre      else if (centre of each circle − *r*) > 0, do       if *x*1 ≤ 1, do       *x*1 = 1       else *x*1 = centre − radius      Repeat 9–16: assign the *y*1 value      Calculate *y*2 using the centre used for *y*1      if (circle centre + *r*) > image row size, do       *y*2 = image row size      else *y*2 = centre + radius      Repeat 19–21: assign the *x*2 value (*x*1 centre and *r*)      Crop image with coordinate (*x*1: *x*2, *y*1: *y*2)      Resize cropped images to 60 × 60 (depending on the classifier input size)   end for end for

### 3.5. Image Feature Extraction

**Figure 6:**
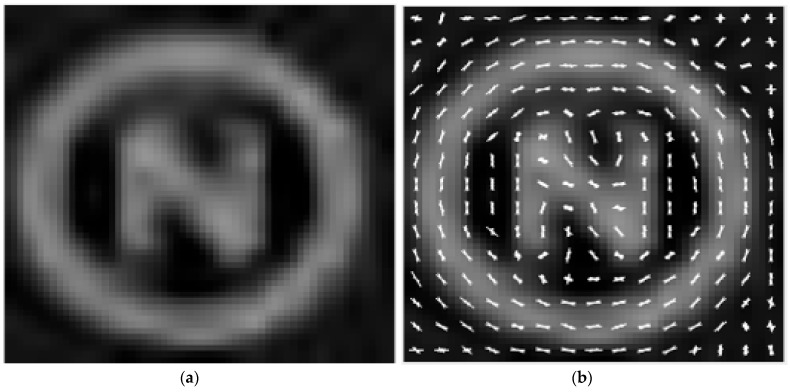
(**a**) Sample marker image. (**b**) [4 × 4] HOG feature extraction.

The marker features are extracted from the OoI and used for training a machine learning classifier. Figure 6 illustrates a sample image, whereby its distinct features are extracted by employing a [4×4]  cell-size Histogram of Orientated Gradient (HOG) feature extractor and are subsequently stored in a feature vector [32]. The choice of using HOG for this application is that it has the following attributes:

- The features allow for a more robust image when subjected to variations in illumination and shading.
- They are relatively invariant to small translations and rotations, which makes them suitable for marker classification in different orientations or positions.
- Unique information about marker edges and corners is inherently encoded.
- Finally, they are computationally efficient when compared to other methods, which would allow for efficient real-time implementation in an embedded system.

The gradient of each pixel is calculated using the HOG feature extraction algorithm, which is governed by Equation (15):

(15) $$G_{h}=\left|G\right|+G_{b}$$

where $G_{h}$ represents the histogram gradient magnitude, and $G_{b}$ denotes the contribution pixel gradient magnitude. $\left|G\right|$ is the absolute gradient magnitude, and the orientation gradient is represented as $\theta_{\left(x,y\right)},$ which was previously defined by Equations (6) and (7), respectively.

Equation (16) is used to calculate the contribution of each pixel gradient magnitude:

(16) $$G_{b}=\frac{\left(\theta_{\left(x,y\right)}-Bin\right)}{BinSize}\times \left|G\right|$$

where Bin is the value obtained next to the orientation angle defined by (x,y). The BinSize is the number of histogram bins as defined by [33] and has a selected size of 20 based on a trade-off between the computational cost and the number of features being extracted.

Equation (17) is the normalization feature vector employed to reduce lighting variations. The created feature vectors, or Bag of Features (BoF), are affected by the gradients of each image since they are sensitive to ambient lighting.

(17) $$V_{L2-norm}=\frac{v}{\sqrt{{{\left\|v\right\|}_{2}}^{2}+\epsilon^{2}}}$$

where $V_{L2-norm}$ is the normalised feature vector. v denotes the unnormalized feature vector. ${\left\|v\right\|}_{2}$ represents the length of vector where L2-norm is used. ϵ denotes the small normalisation constant.

The steps given in Section 3.5 are summarized in the pseudocode as illustrated by Algorithm 3.
**Algorithm 3.** Feature extraction using HOGInput: Cropped images (Algorithm 2) Output: Concatenated feature vector
 Resize cropped images to 60 × 60 Declare vector variables (*trainingFeatures*, *trainingLabels*) Find the number of cropped images (*numCropImages*) for *numCropImages* ≥ 1, do   Divide into a cell   for each cell, do      Obtain HOG for every pixel      Compute the magnitude and orientation using Equations (15) and (16)      Normalize the histogram using Equation (17)   end for   Form BoF (concatenated feature vector) end for

### 3.6. Image Classification

**Figure 7:**
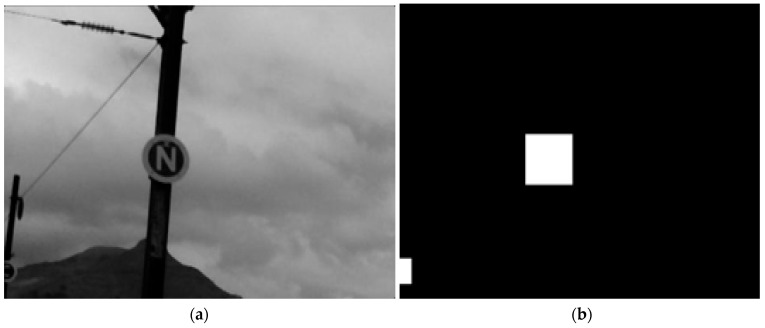
(**a**) Greyscale sample image. (**b**) Ground-truth image.

Prior to training, the images are further processed to create ground-truth images used to validate the accuracy of the model. These ground-truth images were manually created using Paint.net software, and an illustrative example is shown in Figure 7. The delineated RoIs in the white foreground are the ground-truth images of the markers. Classification is then employed in the training stage to output the predicted dataset to validate the segmentation accuracy by comparing it with the ground truth images.

**Figure 8:**
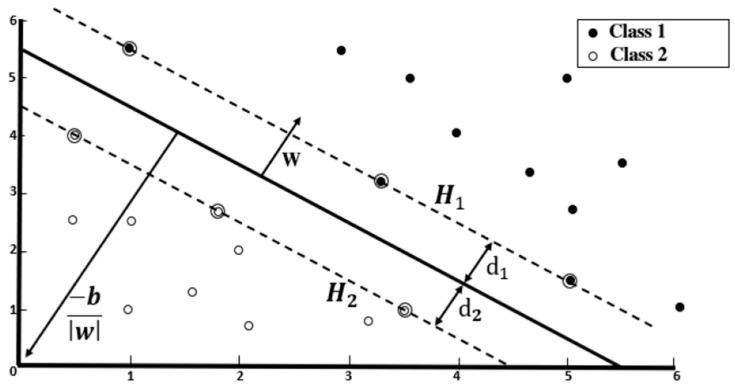
Hyperplane illustrating two linearly separable classes.

**Figure 9:**
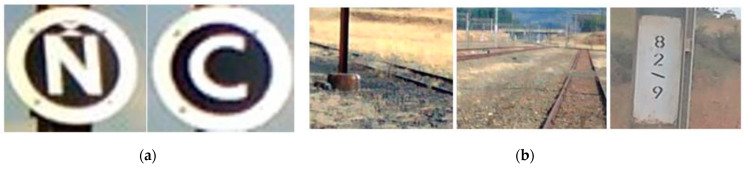
(**a**) Open (‘N’) and Close (‘C’) marker classes. (**b**) Negative image samples.

For image classification, the Linear Support Vector Machine (LSVM) is used. Figure 8 exemplifies a SVM as a classifier that separates two distinct classes by finding the optimal hyperplane with the maximum margin that separates the features. The hyperplane linearly separates these features into their respective classes or labels (“N: Open” and “C: Close”). Figure 9 illustrates captured sample images of the two classes and negative samples.

Equation (18) is the hyperplane function, defined by:

(18) $$w\cdot x_{i}+b=0$$

where w is the weight vector, $x_{i}$ represents the training feature vectors, where i=1,…, L training features and b denotes the bias. The classes closer to the hyperplane are the support vectors and its implementation depends on the selection of w and b such that the training data can be defined as:

(19) $$w\cdot x_{i}+b\;\geq +1\;\;\;\;\;\;\mathrm{for}\;y_{i}=+1$$

(20) $$w\cdot x_{i}+b\;\leq -1\;\;\;\;\;\mathrm{for}\;y_{i}=-1$$

where $y_{i}\;\in \;\left\{-1,+1\right\}$ being the classes for open (“N”) and close (“C”) markers. Equations (19) and (20) can be combined and expressed as:

(21) $$y_{i}\left(w\cdot x_{i}+b\right)-1\geq 0$$

The support vectors $H_{1}$ and $H_{2}$ are thus described by:

(22) $$w\cdot x_{i}+b=+1\;\;\;\;\;\mathrm{for}\;H_{1}$$

(23) $$w\cdot x_{i}+b=-1\;\;\;\;\;\mathrm{for}\;H_{1}$$

The margin is defined as the distance between the support vectors and the hyperplane and are equidistance such that $d_{1}=d_{2}$. The total margin can be expressed as $\frac{2}{\left\|w\right\|}$, whereby the minimum margin region $\frac{1}{2}\;{\left\|w\right\|}^{2}$ is solved by constrained optimization by applying Lagrange multipliers defined as:

(24) $$L_{p}\equiv \frac{1}{2}{\left\|w\right\|}^{2}-\sum_{i=1}^{L}a_{i}y_{i}\left({w\cdot x}_{i}+b\right)+\sum_{i=1}^{L}a_{i}$$

where $a_{i}\geq 0$.

The LSVM image classification is summarized in the pseudocode, as shown by Algorithm 4.
**Algorithm 4.** Image classification training using LSVMInput: Training and Validation BoF (Algorithm 3) Output: Class label for each BoF
 Train the LSVM classifier for any classes {−1, +1}, do   Use Equation (24) to determine the optimal hyperplane. end for for each feature vector in the validation dataset, do   With majority votes, assign the class label end for for each predicted image in the dataset, do   Compare image with ground truth image   Compute the similarity result into one variable end for Calculate the accuracy of the trained model by using the mean score Repeat steps 8 to 12 for model validation

To optimize the LSVM model for the marker image dataset, the MATLAB^®^ Statistics and Machine Learning Toolbox™ was used. Model cross-validation was performed using two-fold and five-fold validation passes to ensure that an accurate model was obtained. Details of the model hyperparameters are shown in Table 1. During the training phase, the optimization algorithm iteratively adjusts the model’s parameters to minimize the classification error and maximize the margin between the classes.

## 4. Results

### 4.1. Optimal Parameter Selection for the Bilateral Filter

The selection of an optimal bilateral filter was made through the comparison of Gaussian spatial weighting ($\sigma_{s})$ and Gaussian range weighting ${(\sigma }_{r})$ parameters. Filters with varying parameters were applied to a greyscale image embedded with noise. The performance of each parameter combination is shown in Table 2. It can be noted that the bilateral filter with weightings of $\sigma_{s}=1$ and $\sigma_{r}=650.25$ are the best parameters for the application when considering correlation performance and computational time as the main selection criteria.

### 4.2. Comparison of Edge Detection Operators

**Figure 10:**
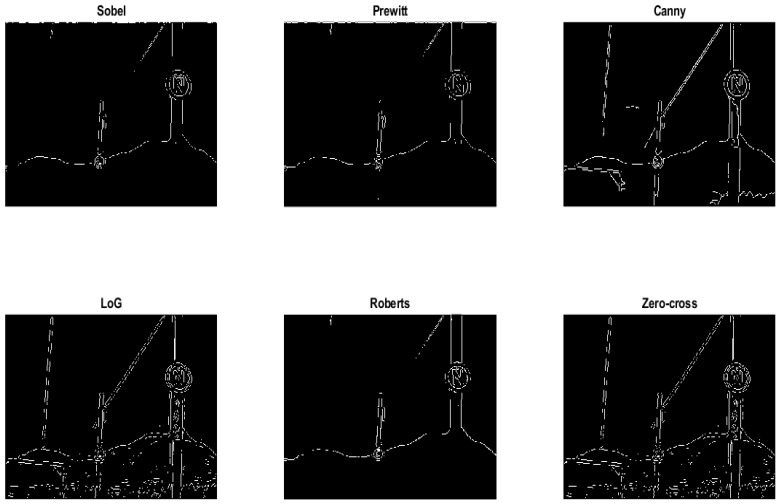
Edge detection comparison of railway signal marker.

The Sobel edge detection was compared to several other methods such as Prewitt, Canny, Laplacian of Gaussian (LoG), Roberts and Zero-cross. Figure 10 illustrates the background subtraction images for each operator. LoG, Zero-cross and Canny methods produced significant background artefacts in the sampled images, while Sobel, Prewitt and Roberts methods showed fewer artifacts but exhibited discontinuous edges. Correlation experiments were performed for each operator and an F1-measure was applied to compute the performance of each operator. Notably, the Sobel method achieved 57.81%, while Canny and Prewitt resulted in 54.51% and 55%, respectively.

### 4.3. Image Classification Results

#### 4.3.1. Evaluation Metric

Performance analysis of the proposed system was conducted using accuracy, precision, recall and F1-score. The accuracy of the methodology has been computed utilizing the numerical details of True Positive (TP), False Positive (FP), True Negative (TN) and False Negative (FN). The details of the marker confusion matrix are given in Table 3.

Model accuracy is defined as:

(25) $$Accuracy=\frac{TP+TN}{TP+TN+FP+FN}$$

Also, precision, recall and F1-score evaluation metrics are respectively calculated as:

(26) $$Precision=\frac{TP}{TP+FP}$$

(27) $$Recall=\frac{TP}{TP+FN}$$

(28) $$F1\;score=\frac{2\times P\times R}{P+R}$$

#### 4.3.2. LSVM Model Performance

Table 4 details the performance of the LSVM classification for the two-fold and five-fold cross-validation during the testing phase. The BoF sizes that were obtained from a [2 × 2] cell size was 43 Mbytes, [4 × 4] cell size resulted in 10 Mbytes, [8 × 8] cell size was 2 Mbytes, and finally, [16 × 16] cell size used 211 Kbytes. The [8 × 8] cell size resulted in the highest precision, indicating the quality of positive predictions made by the model. In both cross-validation cases of the various HOG images, the [4 × 4] cell size resulted in the best overall performance with a recall of 98.63% and 98.21% and an F1 score of 97.52% and 97.99%, respectively.

#### 4.3.3. Efficacy of the System Performance

**Figure 11:**
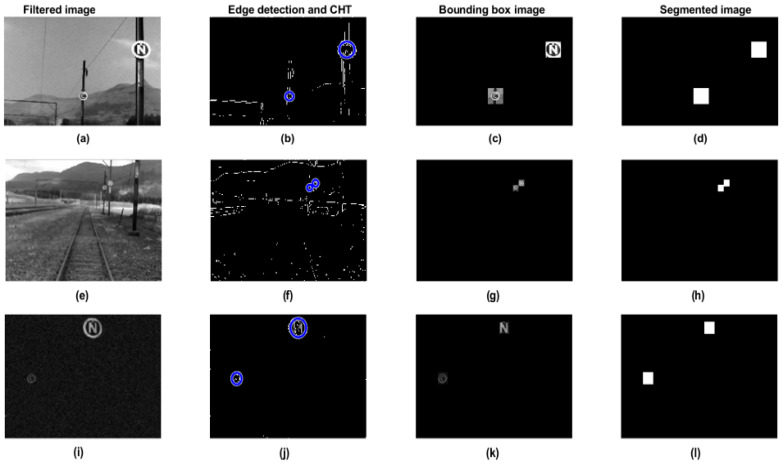
Sample test images for each stage. Images processed for a sunny condition, (**a**–**d**). Images process for a cloudy condition, (**e**–**h**). Images processed for a dark lighting condition, (**i**–**l**).

Figure 11 shows representative image processing for each stage of the proposed system. The classification results are based on three lighting conditions, namely, sunny, cloudy, and dark. Figure 11a,e,i are the filtered images from the bilateral filter during sunny, cloudy, and dark environments. The Sobel edge detector is applied to remove background artifacts, and then the OoI’s are delineated with a CHT, which is illustrated in Figure 11b,f,j in the different sceneries. Figure 11c,g,k are the extrapolated OoI’s from applying a bounding box with the (*x*, *y*) coordinates obtained from the CHT method. The latter illustrates classified and predicted OoI’s (markers); however, in Figure 11d,h,l, the images are used to measure the prediction accuracy of the model during experimental evaluation. The performance of the LSVM image classification was compared to that of other machine learning classification algorithms, namely Decision Tree (DT) CART and ID3, Linear Discriminant Analysis (LDA), Quadratic Discriminant Analysis (QDA), Naïve Bayes, Quadratic SVM (QSVM), Cubic SVM (CSVM), Adaptive Boosting (Adaboost), Convolutional Neural Network (CNN), and finally K-Nearest Neighbors (K-NN). After training, each classification algorithm was validated using a corresponding [4 × 4] HOG cell size, which results in a BoG size of 10 Megabytes. The performance of each classifier is measured by its efficacy during training, validation, and prediction speed and is presented in Table 5. The LSVM during training achieved 93.40% and at validation achieved 94% with a prediction speed of 75 objects per second (obj/sec). The QSVM displayed similar validation results, but detected objects at a lower speed of 68 obj/sec. CNN performance using one layer resulted in 90.4% validation accuracy at a notably higher speed of 82 obj/sec. The Adaboost model resulted in a significantly poor performance of 57.8% during validation. Furthermore, Naïve Bayes and K-NN models displayed relatively lower obj/sec detection speeds.

## 5. Conclusions and Recommendations

The article presents a computer vision model to switch electric locomotives traversing through a neutral section rather than conventional methods. A detailed process, from setting up the model to training and validation, was explored through different techniques. Five steps were proposed for the system to be affected, namely, image acquisition, image pre-processing, image segmentation, feature extraction, and finally classification. For each image, a bilateral filter was utilized to eliminate image noise, while a Sobel operator and CHT were employed to segment RoI. A bounding box extracted OoI’s from the RoI, and a LSVM classification algorithm was then chosen to classify and predict the railway signal markers under different lighting conditions. The HOG feature extractor was chosen based on its performance to effectively describe the unique features of the markers.

While the overall model performance of the system using LSVM may be 94% accurate, it may not be an acceptable result against industrial standards. However, considering the 640 × 480 camera resolution was used for acquiring the dataset images and there is limited literature on computer vision for automatic switching of electric locomotives, it can be considered an acceptable result. However, there is still room for improving the model by increasing the dataset along with higher-resolution images that can provide greater marker clarity at further distances and employing ensemble techniques with other methods to achieve higher model accuracy. Furthermore, a night vision camera could be used to capture images under low lighting conditions.

## Figures and Tables

**Table 1:** LSVM hyperparameter settings in the MATLAB^®^ graphical user interface.

Kernel Function	Kernel Scale	Kernel Offset	Box Constraint Level	Cross Validation Folds
Linear	‘auto’	0	1	2 and 5

**Table 2:** Performance evaluation of Bilateral filter parameters.

Gaussian Weighing		Correlation of the Original Image versus the Filtered Image (%)	Time (msec)
$\sigma_{s}$	$\sigma_{r}$		
1 *	10	99.33	2.70
	30	99.37	2.88
	100	99.41	2.75
	300	99.43	2.75
	650.25 *	99.43	2.62
3	10	99.36	12.37
	30	99.42	11.37
	100	99.46	12.76
	300	99.39	10.73
	650.25	99.26	12.76
10	10	99.36	389.52
	30	99.41	412.96
	100	99.41	444.07
	300	99.43	368.09
	650.25	98.75	326.18

* Optimal parameter selection.

**Table 3:** Marker image confusion matrix.

**Actual** **image**	**Predicted image**			
		Close ‘C’	Negative ‘I’	Open ‘N’
	Close ‘C’	TP	FP	FP
	Negative ‘I’	FN	TP	FN
	Open ‘N’	FN	FN	TP

**Table 4:** Performance analysis of LSVM model for two-fold and five-fold cross-validation.

	Cell Size	Precision (%)	Recall (%)	F1 Score (%)
Two-fold cross-validation	[2 × 2]	93.60	88.79	91.13
	[4 × 4]	96.43	98.63	97.52
	[8 × 8]	96.79	95.91	96.35
	[16 × 16]	95.67	91.28	93.43
Five-fold cross-validation	[2 × 2]	95.96	98.17	97.05
	[4 × 4]	97.77	98.21	97.99
	[8 × 8]	99.09	96.89	97.98
	[16 × 16]	97.16	92.76	94.91

**Table 5:** Performance evaluation of each classification model using [4 × 4] HOG cell.

Classifier	Training Accuracy (%)	Validation Accuracy (%)	Prediction Speed (Objects/Second)
DT (CART)	75.4	80.7	72
DT (ID3)	74.1	77.1	73
LDA	94.7	92.8	78
QDA	85.1	81.9	71
Naïve Bayes	85.1	81.9	26
LSVM	93.4	94.0	75
QSVM	93.9	94.0	68
CSVM	93.0	92.8	74
AdaBoost	57.5	57.8	68
CNN	90.8	90.4	82
K-NN (2) *	82.0	80.7	13
K-NN (10) *	84.6	80.7	14
K-NN (20) *	83.3	90.4	12

* Selected K-values.

## Data Availability

Data will be made available on request.
